# A New Method for Ecological Risk Assessment of Combined Contaminated Soil

**DOI:** 10.3390/toxics11050411

**Published:** 2023-04-26

**Authors:** Qiaoping Wang, Junhuan Wang, Jiaqi Cheng, Yingying Zhu, Jian Geng, Xin Wang, Xianjie Feng, Hong Hou

**Affiliations:** 1 State Key Laboratory of Environmental Criteria and Risk Assessment, Chinese Research Academy of Environmental Sciences, Beijing 100012, China; wangqiaoping20@mails.ucas.ac.cn (Q.W.);; 2 School of Environmental Science and Engineering, Shaanxi University of Science and Technology, Xi’an 710021, China; 3 College of Resources and Environment, Shanxi Agricultural University, Taigu 030801, China; 4 College of Land and Environment, Shenyang Agricultural University, Shenyang 110866, China; 5 School of Environmental Science and Engineering, Nanjing University of Information Science and Technology, Nanjing 210044, China

**Keywords:** combined contaminated soil, toxicity effect index (*EI*), ecological risk probability (*RP*), soil invertebrates

## Abstract

Ecological risk assessment of combined polluted soil has been conducted mostly on the basis of the risk screening value (*RSV*) of a single pollutant. However, due to its defects, this method is not accurate enough. Not only were the effects of soil properties neglected, but the interactions among different pollutants were also overlooked. In this study, the ecological risks of 22 soils collected from four smelting sites were assessed by toxicity tests using soil invertebrates (*Eisenia fetida*, *Folsomia candida*, *Caenorhabditis elegans*) as subjects. Besides a risk assessment based on RSVs, a new method was developed and applied. A toxicity effect index (*EI*) was introduced to normalize the toxicity effects of different toxicity endpoints, rendering assessments comparable based on different toxicity endpoints. Additionally, an assessment method of ecological risk probability (*RP*), based on the cumulative probability distribution of *EI*, was established. Significant correlation was found between *EI*−based *RP* and the *RSV*−based Nemerow ecological risk index (*NRI*) (*p* < 0.05). In addition, the new method can visually present the probability distribution of different toxicity endpoints, which is conducive to aiding risk managers in establishing more reasonable risk management plans to protect key species. The new method is expected to be combined with a complex dose–effect relationship prediction model constructed by machine learning algorithm, providing a new method and idea for the ecological risk assessment of combined contaminated soil.

## 1. Introduction

Studies on the ecological risk assessment of soil are relatively scarce, especially compared with human health risk assessment [1]. The United States Environmental Protection Agency (USEPA) has formulated guidelines for the derivation of ecological risk screening values (*RSV*) and derived the RSVs of some single pollutants. In China, the toxic effects of cadmium, copper, nickel, zinc, lead, arsenic and antimony on crops, vegetables and invertebrates have been investigated and their thresholds in farmland soils have been estimated [2,3,4,5,6,7]. However, China has yet to establish a system for managing soil ecological risks.

**Scheme 1:**
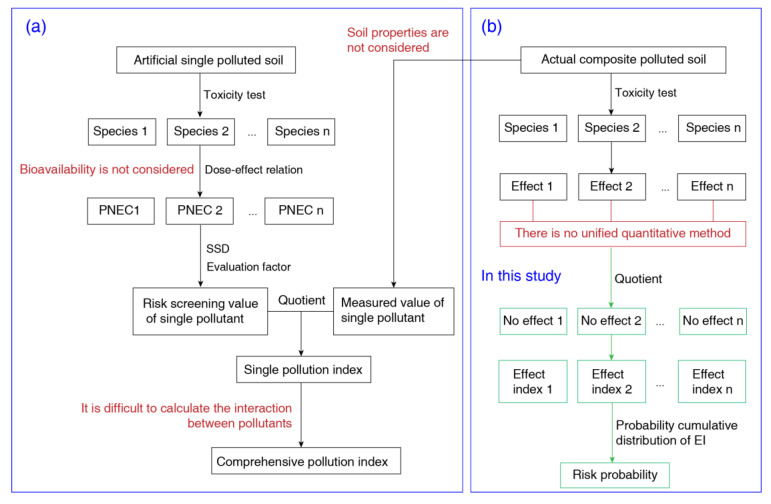
Two methods to assess ecological risk.

RSVs can roughly screen out potential high−risk pollutants in soil. A single risk index (*RI*) based on RSVs is commonly used to characterize the ecological risk of single pollutants, and the Nemerow risk index (*NRI*), based on *RI*, is used to characterize the overall ecological risk of combined pollutants [8,9,10,11,12,13]. However, the deduction process of RSVs neglects the influence of soil properties on toxicity effects, while *NRI* overlooks the complex interaction among different pollutants. Therefore, there is a great deal of uncertainty in the ecological risk assessment of combined contaminated soil by *RI* and *NRI* (Scheme 1a). In fact, most actual sites were contaminated by various pollutants, especially metal smelting, coking and other industries [11,14,15]. Under combined pollution states, complex interactions among pollutants could affect their bioavailability and biotoxicity, making them different from those under a single contamination state. Therefore, these differences should be fully considered in the formulation of risk assessment methods. Although some studies have established the relationship between toxic effects and soil physicochemical properties, and the relationship between toxic effects and bioavailable concentration, such research data are very limited [16,17,18,19,20]. The combined effects of different pollutants have been investigated and concentration addition (CA) and independent action (IA) models were applied in the risk assessment process [21,22,23]. Nevertheless, the actual contaminated soils were of infinite composition, while the joint effect studies cannot be exhaustive.

The real toxicity of actual polluted soil can be evaluated by toxicity tests conducted in the actual polluted soil (Scheme 1b) [24,25]. Normally, whether a soil is at risk to a testing organism at a toxicity endpoint can be determined by comparison with background soil. However, it is difficult to compare the risk of different toxicity endpoints due to the lack of a uniform quantification method [26,27,28].

Given multiple toxicity endpoints, the question of how to comprehensively conduct ecological risk assessment needs further exploration. In this study, toxicity tests using typical invertebrates *Eisenia fetida*, *Folsomia candida* and *Caenorhabditis elegans* as subjects were carried out in 22 actual complex contaminated soils. The purpose of the research was to explore a set of ecological risk assessment methods that can fully consider each of the toxicity endpoints, providing new ideas and schemes for future ecological risk assessment research.

## 2. Materials and Methods

### 2.1. Soils

Twenty−two heavy metal polluted topsoil samples (0–20 cm) were collected from four contaminated sites in the metal smelting industry (BY, DQ, QL, ZS). Soil samples were air−dried, screened (2 mm), and thoroughly mixed before use. The detection methods for soil physicochemical properties and pollutant content are as follows.

#### 2.1.1. Soil pH

Soil pH was measured by potentiometric method using an acidometer (FE28, Mettler Toledo Technology (Shanghai, China) Co., Ltd.).

#### 2.1.2. Organic Matter Content

An amount of 0.05–0.5 g soil was placed into a test tube and 10 mL of 0.4 mol/L K_2_Cr_2_O_7_ sulfuric acid solution was added. After heating in an oil bath at 170–180 °C for 5 min, the solution was transferred to a conical flask and water was added to 50 mL. The excess K_2_Cr_2_O_7_ was titrated with a 0.1 mol/L FeSO_4_ solution. The organic matter (OM) content of soil was calculated based on the consumption of K_2_Cr_2_O_7_.

#### 2.1.3. Clay Content

The determination method of soil clay (d < 0.002 mm) content refers to the pipette method in the Chinese standard HJ 1068−2019 [29].

#### 2.1.4. Cation Exchange Capacity

At (20 ± 2) °C, 3.5 g soil was extracted with a 50.0 mL 1.66 cmol/L Co(NH_3_)_6_Cl_3_ solution, and the cations in the soil were exchanged by Co(NH_3_)_6_Cl_3_ into the solution. The absorbance of the solution was measured at 475 nm using ultraviolet−visible spectrophotometer (UV756, Shanghai Youke Instrument Co., Ltd. in Shanghai, China). The cation exchange capacity (CEC) of the soil was calculated according to the absorbance difference of the leaching solution before and after leaching.

#### 2.1.5. Mn, Fe, and Al Content

An amount of 0.1–0.2 g soil was placed into a closed digestion tank of polytetrafluoroethylene, amounts of 6 mL hydrochloric acid (1.19 g/mL) and 2 mL nitric acid (1.42 g/mL) were then added before being digested with a microwave at 120, 150 and 185 °C for 2, 5 and 40 min respectively, and the total content of Mn, Fe and Al was determined using an inductively coupled plasma mass spectrometer (7900 ICP−MS, Agilent in Tokyo, Japan).

#### 2.1.6. Hg, As and Sb Content

An amount of 0.1–0.5 g soil was placed into a closed digestion tank, amounts of 6 ML of hydrochloric acid (1.19 g/ML) and 2 ML of nitric acid (1.42 g/ML) were then added before being digested with a microwave at 100, 150, and 180 °C for 2, 3 and 25 min respectively. The total content of Hg, As and Sb was determined using an atomic fluorescence spectrophotometer (AFS−8510, Beijing Haiguang Instrument Co., Ltd. in Beijing, China).

#### 2.1.7. Cr, Pb, Cu, Zn and Cd Content

An amount of 0.25–0.5 g soil was placed into a closed digestion tank. Amounts of 6 mL nitric acid (1.42 g/mL), 3 mL hydrochloric acid (1.19 g/mL) and 2 mL hydrofluoric acid (1.16 g/mL) were added in turn before being digested with a microwave at 120, 160 and 190 °C for 3, 3 and 25 min respectively. The total content of Cr, Pb, Cu and Zn was determined with flame atomic absorption spectroscopy (280FS AA, Agilent in Selangor, Malaysia), and determine the total content of Cd with graphite furnace atomic absorption spectrometer (240Z AA, Agilent in Selangor, Malaysia).

### 2.2. Test Organisms

*Eisenia fetida*, bought from Kunlong Farm (Beijing, China), was cultured indoors for at least two weeks before tests and was regularly fed with oats. The temperature was controlled at 20 ± 2 °C, with 16 h of light (light intensity 400–800 lx), 8 h of darkness, and soil water content that was maintained at 50%. Active and similar mature earthworms were selected during the experiment, and their mass was in the range of 300~500 mg. A population of *Folsomia candida*, originally from the Institute of Soil Sciences, Chinese Academy of Sciences, had been cultured in our laboratory for over six years using an artificial climate box (Ningbo Saifu Experimental Instrument Co., Ltd., in Ningbo, China). This population was reared in a moist mixture of gypsum and charcoal at 20 ± 1 °C using a 16−h light/8−h dark light regime and fed small amounts of dry bread yeast twice a week. Distilled water was added weekly to maintain the moisture content of the medium. *Caenorhabditis elegans* (var. Bristol strain N2) and *Escherichia coli* (strain OP50) were purchased from Fujian Shangyuan Biotechnology (Fuzhou, China). *C. elegans* was cultured in Nematode−growth−medium (NGM) agar at 20 ± 1 °C in a constant temperature incubator. *E. coli* OP50 strains were used as the food source of nematode. In order to reduce the influence of individual differences of nematode on the experiment, it was necessary to conduct synchronous culture of nematode before the experiment. See Supplementary Materials for the preparation of NGM agar, *E. coli* culture and synchronous culture of nematode.

### 2.3. Toxicity Tests

#### 2.3.1. *E. fetida* Toxicity Tests

Before toxicity tests, the earthworms were placed in a beaker with wet filter paper and treated with intestinal cleansing in an incubator at 20 °C for 24 h. An amount of 500 g of air−dried soil was placed in a beaker with 5 g of cow dung and an appropriate amount of deionized water. During the entire exposure period, the soil moisture content was maintained within 10% change by weighing and water replenishing. Three days later, 10 earthworms were added to the soil and cultured in an artificial climate box (Ningbo Saifu Experimental Instrument Co., Ltd., Ningbo, China). Other culture conditions were the same as feeding conditions. Three parallel groups were set up in each soil and observed continuously for 56 days. The number of dead earthworms was recorded every day, and the dead earthworms were promptly removed from the beaker [30,31].

#### 2.3.2. *F. candida* Toxicity Tests

An amount of 30 g of air−dried soil and an appropriate amount of deionized water were placed in a culture tank. During the whole exposure period, water was added by weighing method to keep the soil moisture content within a 10% change. After three days, 10 synchronous cultured *F. candida* and a small amount of yeast were added to the soil. Yeast was supplemented once a week, with 6 parallel groups set for each soil. On the 7th and 28th days, 3 parallel groups were taken from each soil sample, and the whole system in the culture tank was poured into a large 1 L beaker. An appropriate amount of deionized water and a few drops of blue ink were then added. The soil suspension was stirred from bottom to top with a glass rod and left to stand for 1–2 min after stirring. After all the adults and larvae were floating on the water surface, photos were taken for preservation. Image J software was used to count the number of surviving adults and newborn larvae.

#### 2.3.3. *C. elegans* Toxicity Tests

An amount of 0.5 g of air−dried soil was placed in the test hole of culture plate, to which was added 100 μL of *E. coli* culture (15 mg·mL^−1^). In order to meet the food and water requirements of *C. elegans* during toxic exposure, the soil water content was adjusted to 80% of the maximum field capacity. Ten first−instar nematode larvae were added to the soil samples through capillary tubes, then the culture plates were sealed and exposed at 20 °C for 96 h without light. Three parallel groups were set for each soil sample. After exposure, 500 μL 0.3 g·L^−1^ acid red 94 (C_20_H_2_C_l4_I_4_Na_2_O_5_) solution was added to the culture plate to dye the cuticle of nematodes. The culture plate was placed in an electric blast drying oven, and the nematodes were killed at high temperature (80 °C) to terminate the toxicity test. The nematodes in the culture plates were recovered by liquid silica suspension and stored in a centrifuge tube at low temperature (4 °C). The recovered nematodes were poured into the petri dish. Under the microscope, the lengths of the nematodes bodies were checked as well as the fertility (if there was at least one egg in the nematode body, it was considered fertile) and the number of larvae.

### 2.4. Ecological Risk Assessment Based on RSVs

The ecological risk of a single pollutant in soil can be reflected by the single ecological risk index (*RI*). The calculation formula is as follows:

(1) $$RI=\frac{C}{RSV}$$

where *RI* is the single ecological risk index, *C* is the measured concentration of single pollutants in soil (mg·kg^−1^), and *RSV* represents the risk screening values in soil (mg·kg^−1^). All adopted RSVs are listed in Table 1.

The Nemerow ecological risk index (*NRI*) can reflect the ecological risk of complex pollutants in soil and highlight the impact of high concentrations of pollutants on ecological risk. The calculation formula is as follows:

(2) $$NRI=\sqrt{\frac{{RI}_{mean}^{2}+{RI}_{max}^{2}}{2}}$$

where *RI_mean_* is the average *RI* and *RI_max_* is the maximum *RI*. *NRI* can be divided into five levels: safe (*NRI* ≤ 0.7); ecological warning line (0.7 < *NRI* ≤ 1.0); low risk (1 < *NRI* ≤ 2.0); medium risk (2.0 < *NRI* ≤ 3.0); and high risk (*NRI* > 3.0).

### 2.5. Ecological Risk Assessment Based on Toxicity Tests

#### 2.5.1. Toxicity Effect Index

Toxicity effect index (*EI*) was defined to represent the toxicity effect of soil on the designated toxicity endpoint of organisms, and the formula is as follows:

(3) $$EI=\frac{E}{NOE}$$

where *E* is the effect of experimental groups and *NOE* is the observed effect of control groups. Specific meanings and values vary by species and toxicity endpoints. In the survival experiment of earthworms/springtails, *E* is the number of living earthworms/springtails after the experiment and *NOE* is the total number of earthworms in the experimental group (*NOE* = 10). In the springtails/nematode propagation test, *E* is the number of larvae in the experimental group and *NOE* is the number of larvae in the control group (springtail *NOE* = 204; nematode *NOE* = 600). In the nematode development experiment, *E* is the number of nematodes with fertility, and *NOE* is the number of nematodes released in the experiment (*NOE* = 10). In the nematode growth test, *E* is the average body length of the experimental group and *NOE* is the average body length of the control group (*NOE* = 1200). In theory, 0 ≤ *EI* ≤ 1; when *EI* = 1, soil has no toxic effect on organisms at all, when *EI* = 0.8, soil has 20% toxic effect on organisms, and so on.

#### 2.5.2. Cumulative Probability Distribution Curve of *EI* and Risk Probability

The *EI* was ranked from smallest to largest to determine its rank R (the rank of the lowest toxicity value is 1, the rank of the second is 2, and so on. If two or more *EI* are the same, they were arranged into a consecutive rank arbitrarily). The cumulative risk probability (*RP*) of *EI* is calculated by the following formula:

(4) $$RP=\frac{R}{N+1}$$

where *R* is the rank of *EI* and *N* is the total number of toxicity endpoints. Using *EI* as the independent variable *X* and the corresponding *RP* as the dependent variable *Y*, the logistic model is used for fitting. The fitting formula is as follows:

(5) $$Y=\frac{1}{1+e^{\frac{P_{1}-X}{P_{2}}}}$$

where *e* is a natural constant and *P*_1_ and *P*_2_ are parameters.

**Figure 1:**
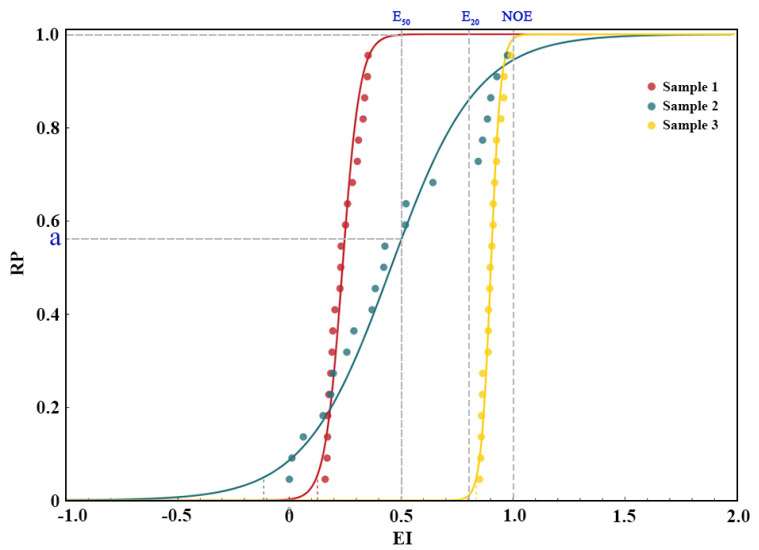
Cumulative probability distribution curve of *EI*—schematic diagram.

To illustrate the model, three groups of rational numbers were randomly generated in the intervals (0, 0.25), (0, 1) and (0.85,1) to simulate the *EI* distribution of the samples of the three soils (Figure 1). Twenty−one numbers in each group represent the *EI* of 21 toxicity endpoints. Samples 1, 2 and 3 simulated the soils of very high, medium and very low risk respectively. The size (range) of *RP* is determined by the position of the curve and the value (range) of *EI*. When determining the risk probability, the risk decision−maker should first set an acceptable safety effect index (*SEI*), and then calculate the *RP* corresponding to the *SEI* according to the curve. If the risk decision maker believes that it is safe for organisms when no toxic effects were observed at all, *SEI* can be set as 1. When the toxicity effect index below 20% is considered to be biosafe, *SEI* = 0.8; If the toxicity effect index less than 50% is considered to be safe for organisms, *SEI* = 0.5, and so on. The soil at the sampling point has a unique and definite risk probability at a definite *SEI* and a given soil sample has a unique and definite risk probability at a definite *SEI*. For example, when *SEI* = 0.5, the *RP* of Sample 2 is a. For soil sample of an extreme high risk (Sample 1) and very low risk (Sample 3), the ecological risk probability can be directly judged without the cumulative probability distribution curve of *EI*: if *EI*_max_ < *SEI*, *RP* = 1; if *EI*_min_ > *SEI*, *RP* = 0.

### 2.6. Data Processing and Statistical Analysis

Two−factor analysis of variance without duplication was performed in Excel 2019 to analyze whether different soils and different toxicity endpoints had significant effects on *EI*. The EEC−SSD software was used to fit the cumulative probability distribution curve, and root mean square error (RMSE) and probability p value of Kolmogorov–Smirnov test (K−S test) were used to evaluate the fitting effect. The closer the RMSE is to 0, the higher the accuracy of model fitting is. If *p* > 0.05, this indicates that the fitting passes the K–S test and the model conforms to the theoretical distribution. The *Hmisc* package of R 4.2.1 software was used to calculate the Spearman correlation coefficient between soil components and the *EI* of different toxicity endpoints, as well as the Pearson and Spearman correlation coefficient between the *EI* of different toxicity endpoints. RStudio software and Origin 2017 software were applied to draw figures and Adobe Illustrator CC 2017 was used to merge images and add annotations.

## 3. Results and Discussion

### 3.1. Ecological Risk Assessment Based on RSVs

Based on the RSVs (Table 1) and the detected concentration of pollutants (Table S1), the *RI* for each of the pollutants in the soil were calculated (Table S2). When *RI* ≥ 1, this indicates that the pollutant had potential risk to soil invertebrates. When *RI* < 1, this shows that the pollutant has no risk to soil invertebrates. Figure S1 illustrates the potential risk pollutants in each sampling site for soil invertebrates. The potential risk pollutants in BY were Zn, Cu, Hg, Pb, Cd, As and Mn. DQ has no potential risk pollutants. The potential risk pollutants in QL were As, Sb, Cr, Zn and Cu. The high potential risk pollutants in ZS are Cu, Zn and As.

**Figure 2:**
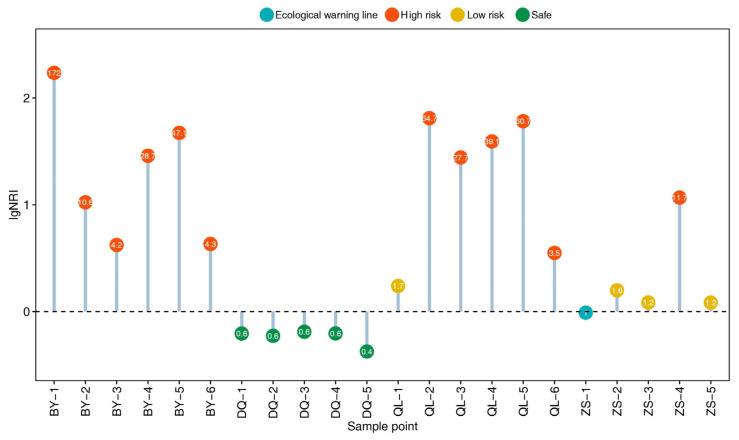
Nemerow risk index of soil samples.

Based on *RI*, *NRI* was calculated (Table S2, Figure 2). All soil samples collected from site BY were at high risk (*NRI* > 3.0). Except QL−1 (low risk, 1.0 < *NRI* ≤ 2.0), all soils from site QL were at high risk (*NRI* > 3.0). One soil sample (ZS−4) from site ZS was at high risk (*NRI* > 3.0), one (ZS−1) was at the warning line (0.7 < *NRI* ≤ 1.0), while the others were at low risk (1.0 < *NRI* ≤ 2.0). All soils samples collected from the site DQ were safe for soil invertebrates.

### 3.2. Ecological Risk Assessment Based on Toxicity Tests

A total of 11 toxicity endpoints were selected (Table S3). The five toxicity endpoints of earthworm were 7−day survival (ES.7D), 14−day survival (ES.14D), 21−day survival (ES.21D), 28−day survival (ES.28D), and 56−day survival (ES.56D). The three toxicity endpoints of springtails were 7−day survival (TS.7D), 28−day survival (TS.28D), and 28−day reproduction (TR.28D). The endpoints of 4−day pregnancy (NP.4D), 4−day reproduction (NR.4D), 4−day body length (NL.4D) were chosen as toxicity endpoints of nematodes. The unreplicated two−factor analysis of variance indicated that soil samples and toxicity endpoints had significant influence on *EI* (*p* < 0.01).

**Figure 3:**
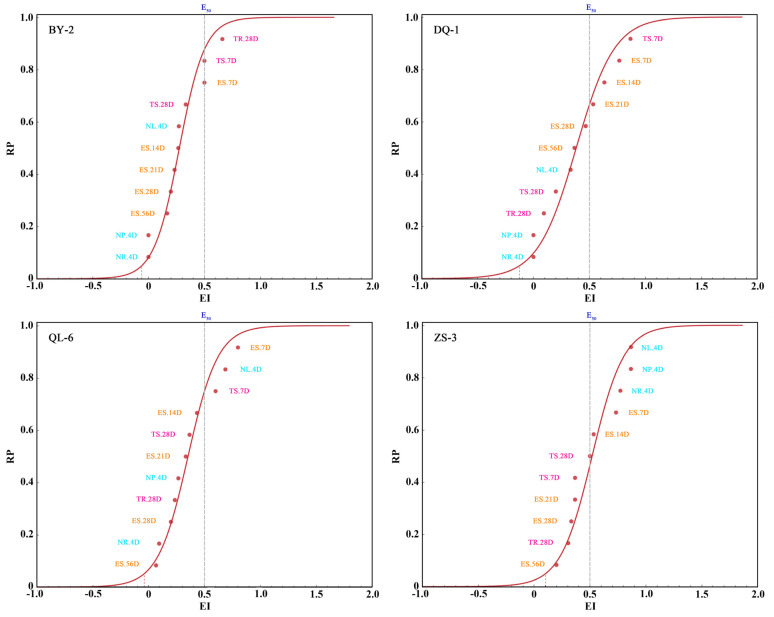
Cumulative probability distribution curve of *EI* for soil BY−2, DQ−1, QL−6 and ZS−3.

The cumulative probability distribution curves of *EI* for all 21 soil samples were fitted (Table 2). Except QL−3, all fitting curves passed the K–S test (*p* > 0.5), the values of RMSE were small, indicating that the probabilistic cumulative distribution model can fit the *EI* distribution of 11 toxicity endpoints at the tested 21 soils samples. *EI*_max_ of QL−3 was less than 0.5, meaning that the soil was extremely toxic to soil invertebrates. Additionally, *RP* of QL−3 can be directly determined as 1. *SEI* was set as 0.5 and the *RP* of each soil was calculated (Table 2). Figure 3 illustrates the *EI* cumulative probability distribution curve of some soil samples, and those of the other soils are shown in supporting materials (Figures S2–S5).

The curve can provide risk managers with three aspects of information:

1. What is the overall risk probability of the soil to the subject organism? For example, *RP* = 0.877 for BY−2 in Figure 3;
2. For which toxicity endpoints was the risk of the soil acceptable or unacceptable? For example, the risk of soil QL−6 for ES.7D, NL.4D and TS.7D was acceptable (*EI* > 0.5), while the risk for other toxicity endpoints was unacceptable (*EI* < 0.5);
3. Relative sensitivity distribution among toxicity endpoints in the risk assessment of soil. For different soil, the sensitivity order of the toxicity endpoints varies among species (Figure 3). For instance, the sensitivity order of the toxicity endpoints in soil BY−2 was NR.4D > ES.28D > TR.28D. While in soil ZS−3, the order was TR.28D > ES.28D > NR.4D. This indicates that if only one toxicity endpoint was used for soil ecological risk assessment, the choice of toxicity endpoint would bring about a great influence on the assessment results.

**Figure 4:**
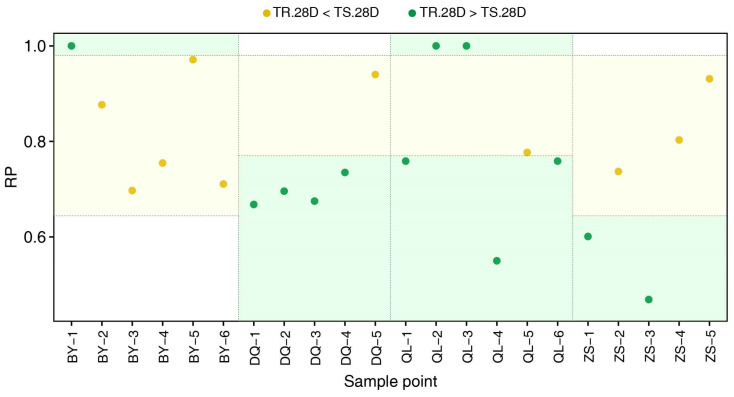
Relationship between *RP* and the sensitivity order of the toxicity endpoints of springtails.

**Figure 5:**
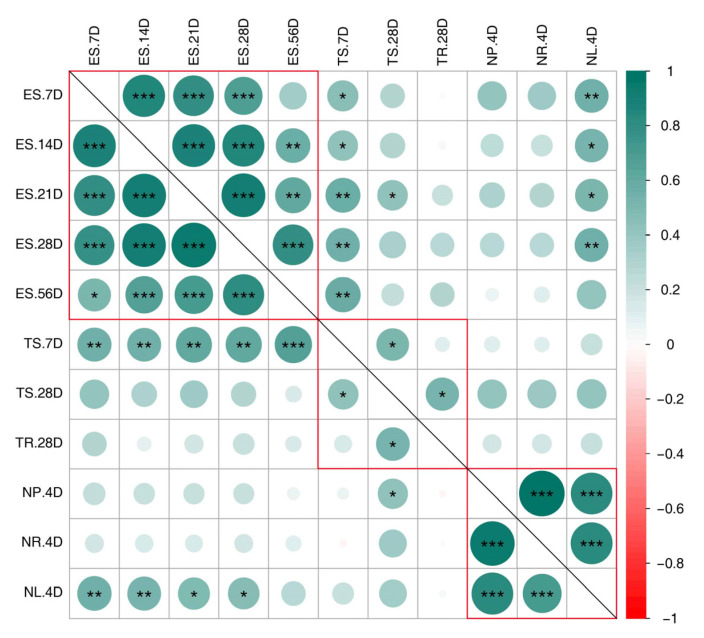
Correlation analysis of toxicity effect index at different toxicity endpoints. The lower−left part is the Pearson correlation, and the upper−right part is the Spearman correlation. (*** *p* < 0.001, ** 0.001 < *p* < 0.01, * 0.01 < *p* < 0.05).

The order of sensitivity among toxicity endpoints of the same species was related to the mechanism of toxicity endpoints. It has been widely recognized that the longer earthworms are exposed to contaminated soil, the more significant the toxic effect of contaminated soil on the earthworms will be. Therefore, the sensitivity order of earthworm toxicity endpoints at any sampling point is ES.56D > ES.28D > ES.21D ≥ ES.14D > ES.7D. The sensitivity order of nematodes was also NR.4D ≥ NP.4D ≥ NL.4D for any soil. Because nematodes do not begin to lay eggs until they undergo four molts and reach 1060 μm in length [39], if a nematode cannot reach the required growth stage, fertility inhibition of the nematode will be affected by both reproductive destruction and growth inhibition. Similarly, if nematode fertility is inhibited, reproductive inhibition of nematodes will be affected by both egg damage and fertility inhibition. There was no obvious regularity in the order of sensitivity of the three toxicity endpoints (TS.7D, TS.28D and TR.28D), but the relative sensitivity of TS.28D and TR.28D seemed to be related to *RP* (Figure 4). When *RP* of soil is low, the sensitivity order of the toxicity endpoint is TR.28 > TS.28. Presumably, the springtails would preferentially adapt to the harsh environment and maintain their own survival after experiencing low toxic stress in soil, delaying life activities such as spawning, but the reproductive ability of springtails did not lose at this time. When the *RP* of the soil was moderate, the sensitivity order of the toxicity endpoint was TR.28D < TS.28D. Possibly, higher toxic soil threatened the survival of the springtails, triggering their emergency reproductive strategy and producing more offspring per adult than in a no toxic stress environment. When the *RP* of soil was very high, the sensitivity order of toxicity endpoints was TR.28 > TS.28. Soil with extremely high toxicity could cause reproductive loss of the springtails before death. Pearson correlation and Spearman correlation analyses showed that there was a significant positive correlation between the toxicity endpoints of the same species (*p* < 0.05) (Figure 5), which was consistent with those presented in the sensitivity distribution curve.

### 3.3. Comparative Analysis between NRI and RP

**Figure 6:**
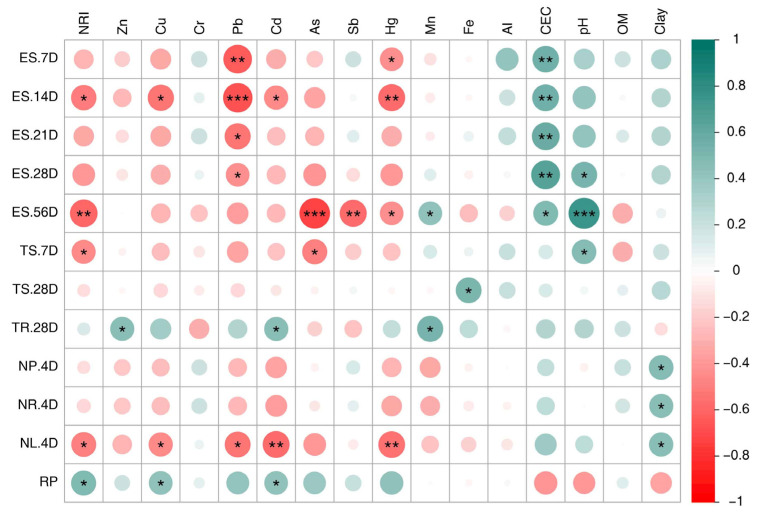
Spearman correlation analysis between toxic effects and soil composition. (*** *p* < 0.001, ** 0.001 < *p* < 0.01, * 0.01 < *p* < 0.05).

Spearman correlation analyses were conducted between *NRI* and *RP* (Figure 6). *NRI* and *RP* were significantly positive correlated (*p* < 0.05). In general, the assessment results of *NRI* and *RP* were basically consistent, and it is feasible to use the cumulative probability distribution method based on *EI* to assess ecological risks.

There is no correlation between *NRI* and *EI* for most single toxicity endpoints, which may be attributed to the following reasons. First, the stress to invertebrates in the combined contaminated soil was not only formed from the nine concerned pollutants, other components and the soil physicochemical properties may also have made an impact [40]. Second, although pollutants are the main source of soil toxicity, *NRI* neglects the interaction among pollutants and the bioavailability of pollutants, possessing great indeterminacy in the risk assessment of soil [41,42,43]. Spearman correlation analysis suggests that the survival of earthworms might be affected by soil CEC and pH (*p* < 0.05). The growth, pregnancy and reproduction of nematodes may be affected by clay content (*p* < 0.05). The survival of springtails may be affected by Fe content in soil (*p* < 0.05). In future toxicity studies of earthworms, nematodes and springtails, more attention needs to be paid to these soil indexes.

**Figure 7:**
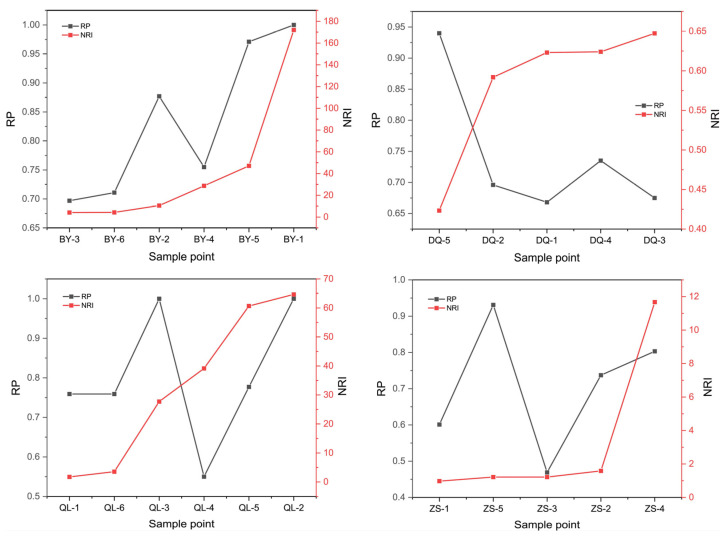
Comparison between *NRI* and *RP* of each soil sample.

*NRI* and *RP* at four sites were further compared and analyzed (Figure 7). In site BY with high *NRI*, *RP* increases with the increase of *NRI*, which is consistent with the result of Spearman correlation analysis (Figure 6). However, the risk probability of BY−2 is higher than expected. In site DQ with low *NRI*, *RP* decreased with the increase of *NRI*, which was contrary to the result of Spearman correlation analysis (Figure 6). Possibly, lower concentrations of pollutants could be beneficial to soil invertebrates, or other soil factors may have exhibited greater effect on invertebrates than pollutants. In site QL with wide span of *NRI*, there was no monotone correlation between *NRI* and *RP*. The toxicity of QL−3 was greater than that expected by *NRI*, while the toxicity of QL−4 and QL−5 was much less than that expected by *NRI*. In site ZS, there is no monotone correlation between *NRI* and *RP*, and the toxicity of ZS−5 is much higher than that expected by *NRI*. The above results further indicate that the correlation between *NRI* and *RP* may be affected by soil components and physicochemical properties [44].

**Figure 8:**
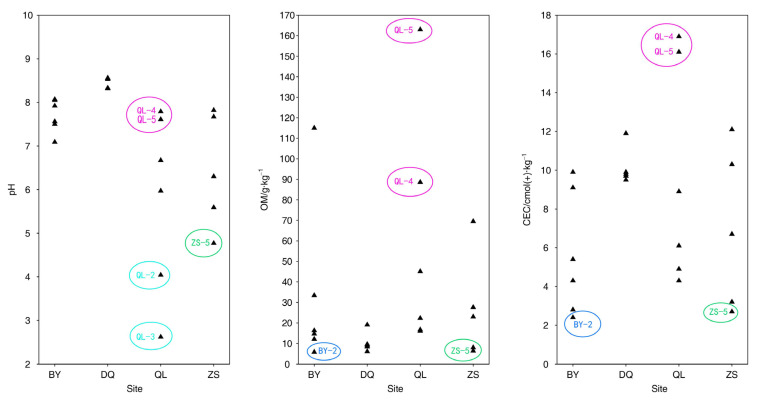
Comparison of physicochemical properties of soil samples.

Several studies have demonstrated that soils with higher pH and soil OM content exhibited less toxicity to earthworms and springtails [16,26,45,46,47]. Toxicity studies on nematodes also found that higher CEC and soil OM content were related to low toxicity [39]. The pH, OM and CEC of each soil sample collected from the four sites were significantly different (Table S4 and Figure 8), which was likely to have a great impact on the ecological risk of soil. For example, the high risk of BY−2 may be influenced by the low CEC and OM. The high risk of QL−2 and QL−3 may be affected by the lower pH. The lower risk of QL−4 and QL−5 may be related to the higher OM, pH, and CEC, and low CEC, OM and pH may have had an impact on the high risk of ZS−5. Therefore, the risk assessment based on the *EI* cumulative probability distribution method could essentially reflect the toxicity of soil to invertebrates, especially the actual combined contaminated soil. The complex interactions among soil pollutants and the effect of soil physicochemical properties were fully taken into consideration. Besides pollutant content, extreme soil physicochemical properties may also have a decisive influence on the life history of soil invertebrates. Taking these factors into account, the risk assessment result could be more convincing in the characterization of soil toxicity. Furthermore, accurate risk assessment could guide risk managers to formulate effective and customized remediation strategies.

### 3.4. Prospects for Risk Assessment Methods

**Scheme 2:**
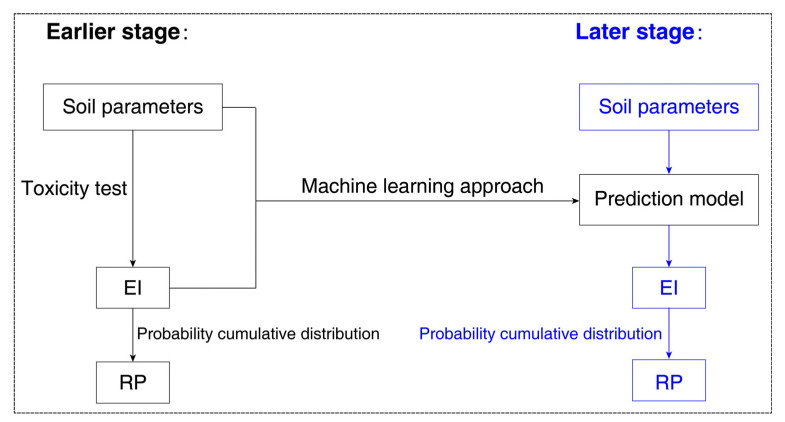
The application of a machine learning model in probabilistic ecological risk assessment.

Although the ecological risk assessment based on toxicity test is reliable, the cost of toxicity tests is high and the cycle is long. Therefore, in the long run, it is still the most economical and effective method to predict the ecological risk from pollutant content while taking the physicochemical properties of soil into account. However, it is very difficult to use traditional statistical methods to study the correlation between soil composition and toxic effects [48,49]. It has been shown that machine learning algorithms can make acceptable predictions about the relationship between responses and multiple factors. In order to reveal the superposition or confrontation of multiple factors, Yu et al. [50] established a tree−structure−based random forest feature importance and feature interaction network analysis framework (TBRFA), and successfully predicted the microbial composition of global soils under 18 environmental factors [51]. Machine learning methods also show great promise in the construction of prediction models of complex dose–effect relationships, if enough toxicity data of combined contamination are available. Therefore, the use of actual pollutant soil for future toxicity tests is recommended, as is the provision of original tabular data, so as to build an open database of composite pollution and toxic effects and to facilitate the prediction of dose–effect relationships by machine learning methods (earlier stage in Scheme 2). The following soil parameters should be considered: the total content of major pollutants, CEC, pH, and OM. In addition, accurate Latin names of species, trial duration, toxicity endpoint and toxicity effect index (*EI*) should be provided. Once the prediction model is established and validated, the toxicity effect index of a specific toxicity endpoint of soil can be predicted without further toxicity tests (later stage in Scheme 2). Finally, based on *EI* predicted by the model, the cumulative probability distribution method can be used to assess the ecological risk of any combined contaminated soil.

## 4. Conclusions

Toxicity effects risk index (*EI*) can normalize toxicity effects of different species at different toxicity endpoints (0 ≤ *EI* ≤ 1). Compared with *NRI*, the cumulative probability distribution curve of *EI* could present more information about toxicity endpoints, which is more practical for risk managers and decision makers. The applicability of the method was preliminarily validated using 11 toxicity endpoints of *E. fetida*, *F. candida*, and *C. elegans* in actual combined contaminated soil. Whether this method can be applied to ecological risk assessment of other species still needs to be verified by toxicity tests, including soil animals, plants, microorganisms and soil enzymes. It might make more sense to use native representative species. This new method is more practical for composite contaminated soils with pollution levels close to RSVs. For pollution levels where all pollutants are far above or far below RSVs, the risk of soil to organisms is foreseeable. Although this method is applicable, considering the cost of risk assessment, it may not be necessary to use this method.

## Figures and Tables

**Table 1:** Risk screening values of soil invertebrates (mg·kg^−1^) [32,33,34,35,36,37,38].

Zn	Cu	Cr	Pb	Cd	As	Sb	Hg	Mn
120 ^1^	80 ^1^	150 ^2^	1700 ^1^	140 ^1^	25 ^2^	78 ^1^	1.3 ^2^	450 ^1^

^1^ Data referenced from the US Environmental Protection Agency. ^2^ Data referenced from China’s “Soil environmental quality Risk control standard for soil contamination of agricultural land”.

**Table 2:** *EI* cumulative probability distribution curve fitting data of soils.

Sample Point	RMSE	*p* (K−S)	*RP* (*EI* = 0.5)	Sample Point	RMSE	*p* (K−S)	*RP* (*EI* = 0.5)
BY−1	0.098	>0.05	1.000	QL−1	0.061	>0.05	0.759
BY−2	0.060	>0.05	0.877	QL−2	0.154	>0.05	1.000
BY−3	0.075	>0.05	0.697	QL−3	0.230	<0.05	1.000
BY−4	0.079	>0.05	0.755	QL−4	0.061	>0.05	0.550
BY−5	0.051	>0.05	0.971	QL−5	0.053	>0.05	0.777
BY−6	0.057	>0.05	0.711	QL−6	0.058	>0.05	0.759
DQ−1	0.054	>0.05	0.668	ZS−1	0.090	>0.05	0.601
DQ−2	0.068	>0.05	0.696	ZS−2	0.048	>0.05	0.737
DQ−3	0.097	>0.05	0.675	ZS−3	0.086	>0.05	0.469
DQ−4	0.078	>0.05	0.735	ZS−4	0.080	>0.05	0.803
DQ−5	0.068	>0.05	0.940	ZS−5	0.068	>0.05	0.931

## Data Availability

The data presented in this study are available in this article and Supplementary Materials.

## Supplementary Materials

The following supporting information can be downloaded at https://www.mdpi.com/article/10.3390/toxics11050411/s1, Table S1: The concentration of heavy metals in soil samples; Table S2: Individual risk index (*RI*) and Nemerow risk index (*NRI*) of soil samples; Table S3: Toxicity effect index (*EI*) of soils; Table S4: Physicochemical properties of soils; Figure S1: Individual ecological risk index of soils based on risk screening values; Figure S2: *EI* cumulative probability distribution of soils collected at BY; Figure S3: *EI* cumulative probability distribution of soils collected at DQ; Figure S4: *EI* cumulative probability distribution of soils collected at QL; Figure S5: *EI* cumulative probability distribution of soils collected at ZS.
