# Supplementary material for: A New Method for Ecological Risk Assessment of Combined Contaminated Soil
Source: Toxics. 2023 Apr 26;11(5):411. doi: 10.3390/toxics11050411 (PMC10220646; doi:10.3390/toxics11050411)
Supplement: Supplementary file 1 [file toxics-11-00411-s001.zip › supplementary materials.pdf]

A new method for ecological risk assessment of complex contaminated site

Qiaoping Wang [1], Hong Hou [1,*], Junhuan Wang [1], Yingying Zhu[1,2], Xin Wang[1,3], Xianjie Feng[1,4] and Jiaqi Cheng[1,5]

[1] State Key Laboratory of Environmental Criteria and Risk Assessment, Chinese Research Academy of Environmental Sciences, 100000 Beijing, China

[2] College of Resources and Environment, Shanxi Agricultural University, 030031 Taiyuan, China

[3] College of Land and Environment, Shenyang Agricultural University, 110866 Shenyang, China

[4] Nanjing University of information Science and Technology, 210044 Nanjing, China

[5] Shaanxi University of Science and Technology, 710021 Xi'an, China

* Correspondence: houhong@craes.org.cn

Preparation of NGM Agar:

17 g·L^{-1} agar powder, 2.5 g·L^{-1} casein peptone and 3 g·L^{-1} NaCl were mixed with deionized water. Autoclave at 121 ℃ for 30 min. Add the following sterile solutions, 1 mL 1 mol·L^{-1} $CaCl_2$, 1 mL 1 mol·L^{-1} $MgSO_4$, 25 mL 1 mol·L^{-1} KH_2PO_4 (pH 6.0±0.2 adjusted with KOH) and 1 mL 5 g·L^{-1} cholesterol ethanol solution. Fill with sterile water to 1000 mL and thoroughly mix, then pour into petri dish to cool and set aside.

Preparation of *E. coli* culture:

Luria-Bertani (LB) medium is composed of 10 g·L^{-1}casein peptone, 5 g·L^{-1} yeast extract and 10g·L^{-1}NaCl. Autoclave at 121 ℃ for 30 min. 17 g·L^{-1} agar powder was added for solid medium. Pick single clone of *E. coli* continuously cultured in solid LB medium, inoculate in liquid LB medium and incubate in rotary shaker (150r·min^{-1}) at 37 ℃ for 14 h. The result *E. coli* solution was used for nematodes culture and toxicity tests.

Synchronous culture method of *C. elegans*:

When a large number of ovulatory adults were distributed on the surface of NGM Agar, *C. elegans* were washed into a centrifuge tube with M9 buffer (33.71 mmol·L^{-1} Na_2HPO_4, 22 mmol·L^{-1} KH_2PO_4, 85.56 mmol·L^{-1} NaCl and 1 mmol·L^{-1}$MgSO_4$). Wash away the remaining *E. coli* on the surface of *C. elegans*. Add lysate (2.5 mol·L^{-1} NaOH and 5% NaClO) to split *C. elegans* to obtain eggs. Wash the eggs with M9 buffer to remove the excess lysate. Culture the eggs in M9 buffer for 14 h, and then the age-synchronized first stage *C. elegans* larvae were obtained.

Table S1. The concentration of heavy metals in soil samples.

Sample point	Mn mg/kg	Zn mg/kg	Cu mg/kg	Cr mg/kg	Pb mg/kg	Cd mg/kg	As mg/kg	Sb mg/kg	Hg mg/kg
BY-1	585	28600	1480	70	24200	654	250	NA	30
BY-2	633	1730	138	74	585	228	18.3	NA	5.49
BY-3	577	652	154	72	737	65.9	30.7	NA	5.57
BY-4	586	4730	629	72	4020	327	135	NA	5.46
BY-5	540	7800	449	66	5950	141	74.4	NA	18.5
BY-6	545	699	107	49	586	42.7	26.4	NA	0.831
DQ-1	345	84	28	96	34	0.08	6.55	NA	0.033
DQ-2	337	50	17	91	31	0.02	8.5	NA	0.045
DQ-3	379	49	18	71	36	0.07	7.22	NA	0.032
DQ-4	362	41	18	91	26	0.08	7.18	NA	0.038
DQ-5	244	43	14	38	33	0.07	5.98	NA	0.035
QL-1	59.8	47	65	108	22	0.18	58.6	15.6	0.234
QL-2	17.1	30	57	261	852	0.22	2230	2690	1.36
QL-3	11.6	16	81	168	113	0.31	953	1300	0.77
QL-4	76.5	29	22	84	40	0.45	140	4250	0.896
QL-5	238	204	66	133	105	2.19	388	6570	0.535
QL-6	191	138	110	100	36	0.28	38.7	370	0.547
ZS-1	167	66	36	74	114	0.04	32	NA	0.079
ZS-2	866	176	141	83	85	0.46	36	NA	0.113
ZS-3	191	65	46	79	65	0.07	40.3	NA	0.146
ZS-4	515	1510	1260	104	140	0.89	26.7	NA	0.182
ZS-5	264	119	47	77	76	0.17	39.4	NA	0.1

Table S2. Individual Risk Index (RI) and Nemerow Risk Index (NRI) of soil samples

Sample point	PI									NRI
	Mn	Zn	Cu	Cr	Pb	Cd	As	Sb	Hg	
BY-1	1.30	238.33	18.50	0.47	14.24	4.67	10.00	0	23.08	172.02
BY-2	1.41	14.42	1.73	0.49	0.34	1.63	0.73	0	4.22	10.56
BY-3	1.28	5.43	1.93	0.48	0.43	0.47	1.23	0	4.28	4.21
BY-4	1.30	39.42	7.86	0.48	2.36	2.34	5.40	0	4.20	28.75
BY-5	1.20	65.00	5.61	0.44	3.50	1.01	2.98	0	14.23	47.13
BY-6	1.21	5.83	1.34	0.33	0.34	0.31	1.06	0	0.64	4.30
DQ-1	0.77	0.70	0.35	0.64	0.02	0.00	0.26	0	0.03	0.62
DQ-2	0.75	0.42	0.21	0.61	0.02	0.00	0.34	0	0.03	0.59
DQ-3	0.84	0.41	0.23	0.47	0.02	0.00	0.29	0	0.02	0.65
DQ-4	0.80	0.34	0.23	0.61	0.02	0.00	0.29	0	0.03	0.62
DQ-5	0.54	0.36	0.18	0.25	0.02	0.00	0.24	0	0.03	0.42
QL-1	0.13	0.39	0.81	0.72	0.01	0.00	2.34	0.20	0.18	1.74
QL-2	0.04	0.25	0.71	1.74	0.50	0.00	89.20	34.49	1.05	64.66
QL-3	0.03	0.13	1.01	1.12	0.07	0.00	38.12	16.67	0.59	27.71
QL-4	0.17	0.24	0.28	0.56	0.02	0.00	5.60	54.49	0.69	39.14
QL-5	0.53	1.70	0.83	0.89	0.06	0.02	15.52	84.23	0.41	60.67
QL-6	0.42	1.15	1.38	0.67	0.02	0.00	1.55	4.74	0.42	3.55
ZS-1	0.37	0.55	0.45	0.49	0.07	0.00	1.28	0	0.06	0.98
ZS-2	1.92	1.47	1.76	0.55	0.05	0.00	1.44	0	0.09	1.58
ZS-3	0.42	0.54	0.58	0.53	0.04	0.00	1.61	0	0.11	1.22
ZS-4	1.14	12.58	15.75	0.69	0.08	0.01	1.07	0	0.14	11.67
ZS-5	0.59	0.99	0.59	0.51	0.04	0.00	1.58	0	0.08	1.22

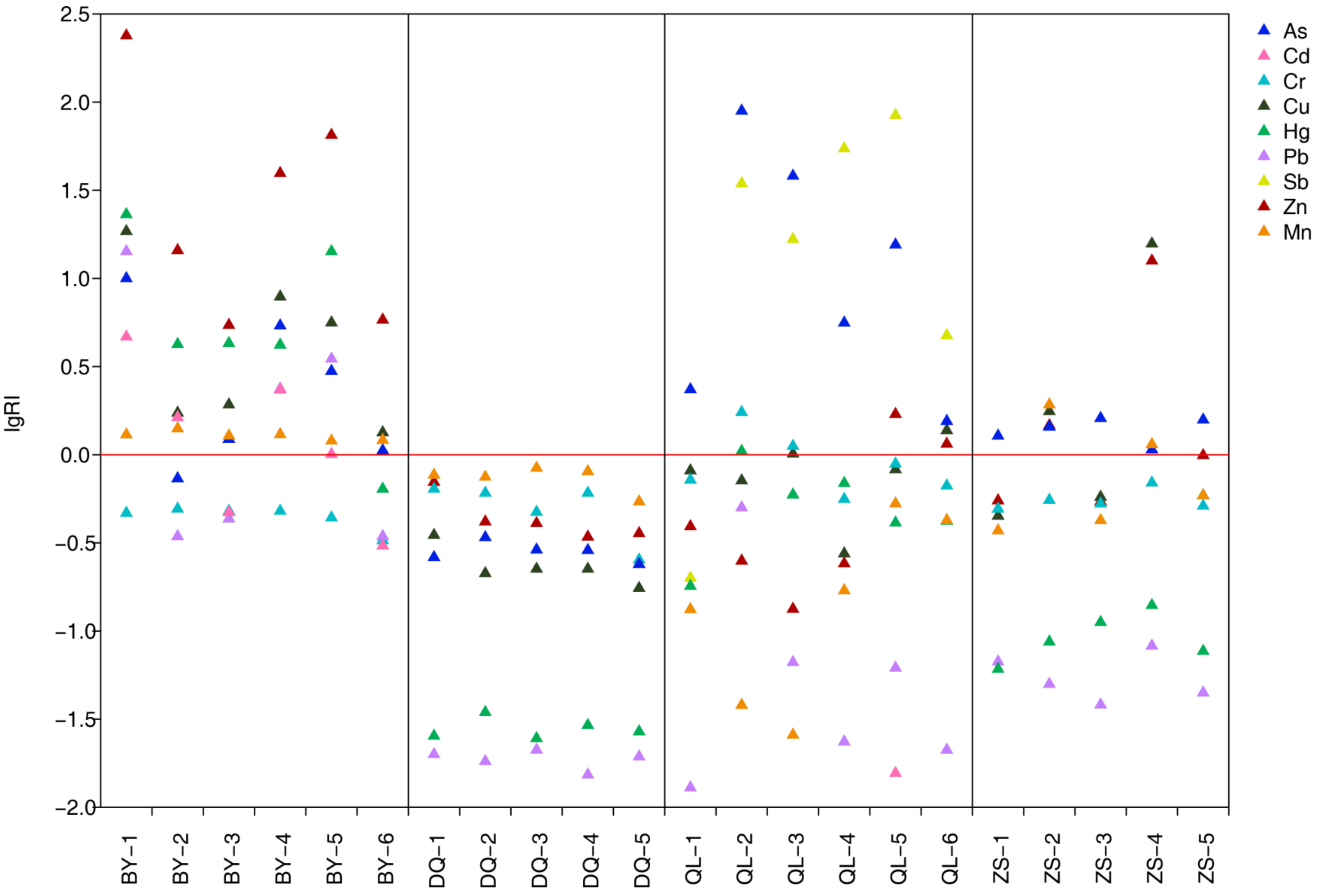

Figure S1. Individual ecological risk index of soils based on risk screening values

Table S3. Toxicity effect index (EI) of soils

Sample point	Toxic end point											
	**ES.7D**	**ES.14D**	**ES.21D**	**ES.28D**	**ES.56D**	**TS.7D**	**TS.28D**	**TR.28D**	**NP.4D**	**NR.4D**	**NL.4D**	**EImax**
BY-1	0.10	0.10	0.10	0.10	0.10	0.30	0.07	0.01	0.00	0.00	0.21	0.30*
BY-2	0.50	0.27	0.23	0.20	0.17	0.50	0.33	0.66	0.00	0.00	0.27	0.66
BY-3	0.63	0.40	0.37	0.37	0.23	0.77	0.30	0.78	0.00	0.00	0.37	0.78
BY-4	0.60	0.27	0.20	0.20	0.10	0.47	0.73	0.95	0.00	0.00	0.22	0.95
BY-5	0.70	0.17	0.13	0.10	0.07	0.33	0.13	0.25	0.00	0.00	0.26	0.70
BY-6	0.67	0.43	0.33	0.27	0.20	0.53	0.50	0.66	0.00	0.00	0.46	0.67
DQ-1	0.77	0.63	0.53	0.47	0.37	0.87	0.20	0.09	0.00	0.00	0.33	0.87
DQ-2	0.77	0.67	0.50	0.43	0.33	0.57	0.23	0.01	0.00	0.00	0.47	0.77
DQ-3	0.90	0.63	0.33	0.33	0.20	0.73	0.23	0.17	0.20	0.04	0.69	0.90
DQ-4	0.73	0.40	0.30	0.23	0.23	0.60	0.47	0.38	0.23	0.18	0.70	0.73
DQ-5	0.60	0.40	0.17	0.17	0.13	0.27	0.17	0.26	0.00	0.00	0.44	0.60
QL-1	0.83	0.50	0.37	0.27	0.10	0.67	0.47	0.11	0.00	0.00	0.40	0.83
QL-2	0.00	0.00	0.00	0.00	0.00	0.30	0.20	0.07	0.00	0.00	0.22	0.30*
QL-3	0.00	0.00	0.00	0.00	0.00	0.00	0.00	0.00	0.00	0.00	0.24	0.24*
QL-4	0.87	0.70	0.60	0.50	0.17	0.47	0.47	0.43	0.23	0.07	0.70	0.87
QL-5	0.80	0.57	0.43	0.27	0.07	0.30	0.37	0.48	0.10	0.02	0.44	0.80
QL-6	0.80	0.43	0.33	0.20	0.07	0.60	0.37	0.24	0.27	0.09	0.69	0.80
ZS-1	0.60	0.37	0.27	0.23	0.13	0.60	0.63	0.30	0.70	0.35	0.82	0.82
ZS-2	0.77	0.60	0.33	0.33	0.23	0.47	0.30	0.53	0.00	0.00	0.44	0.77
ZS-3	0.73	0.53	0.37	0.33	0.20	0.37	0.50	0.31	0.87	0.77	0.87	0.87
ZS-4	0.70	0.37	0.30	0.30	0.17	0.20	0.07	0.53	0.23	0.16	0.72	0.72
ZS-5	0.67	0.47	0.20	0.20	0.07	0.23	0.17	0.23	0.00	0.00	0.39	0.67

*$EI_{max} < 0.5$

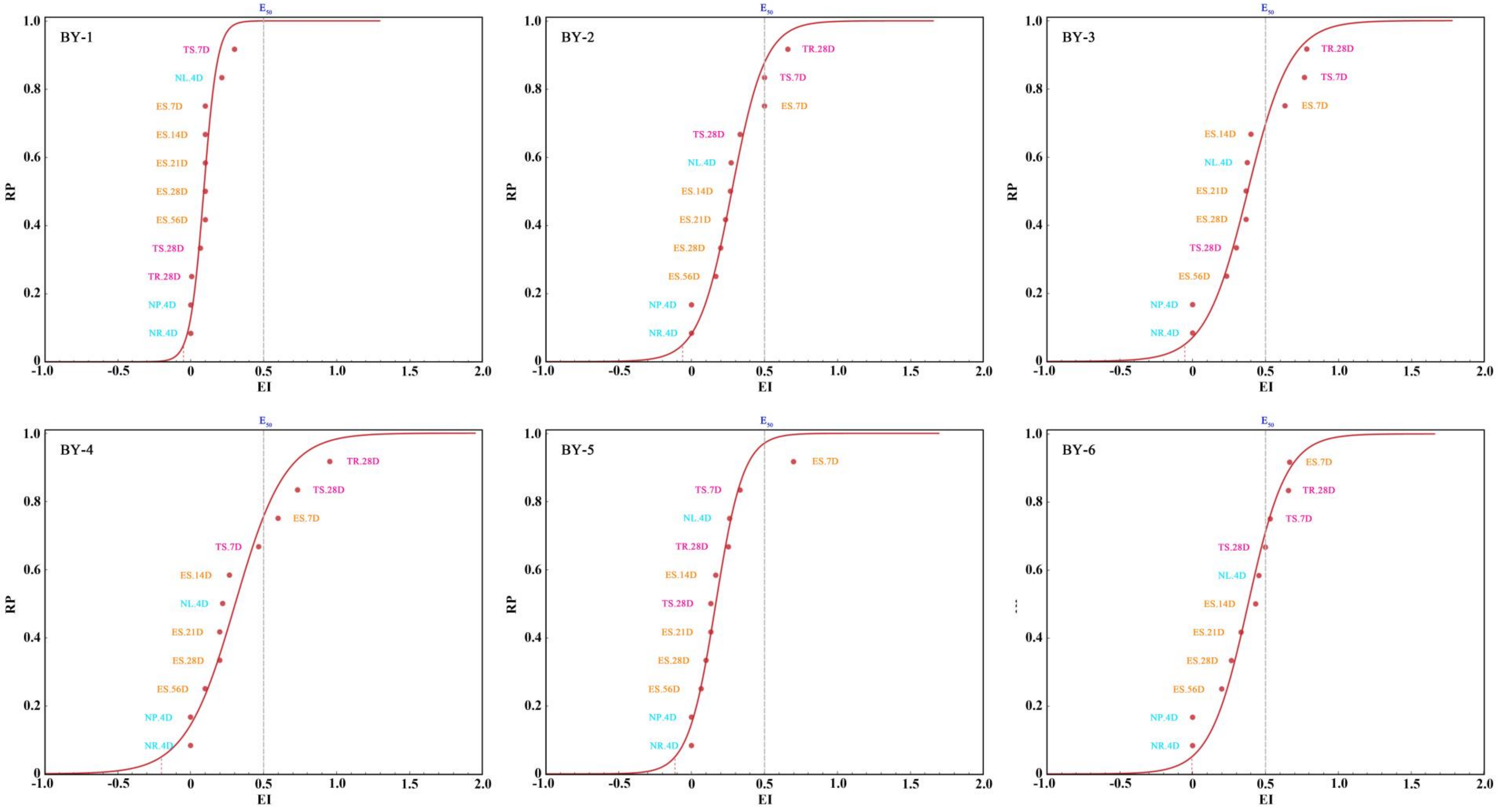

Figure S2. EI cumulative probability distribution of soils collected at BY

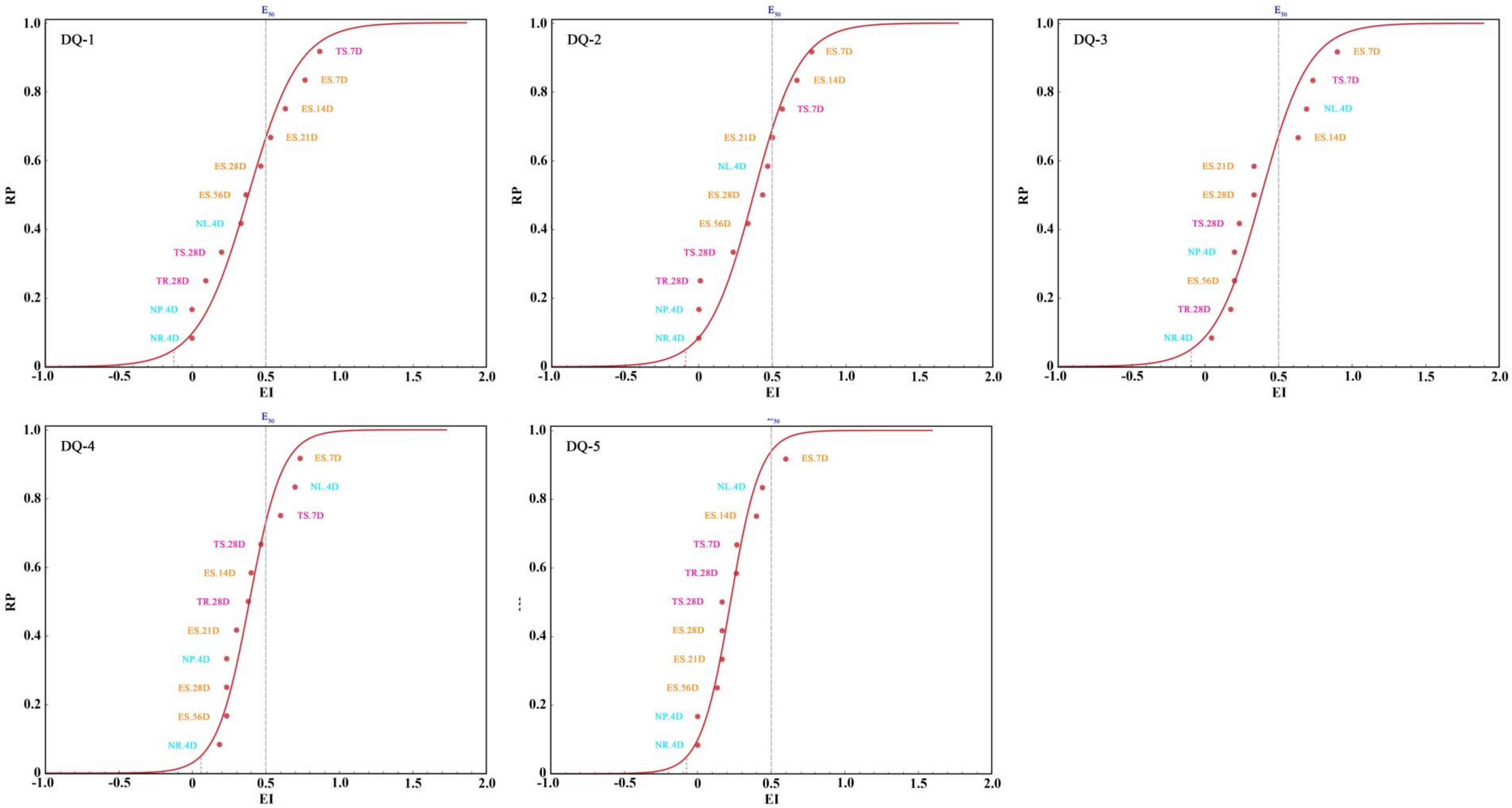

Figure S3. EI cumulative probability distribution of soils collected at DQ

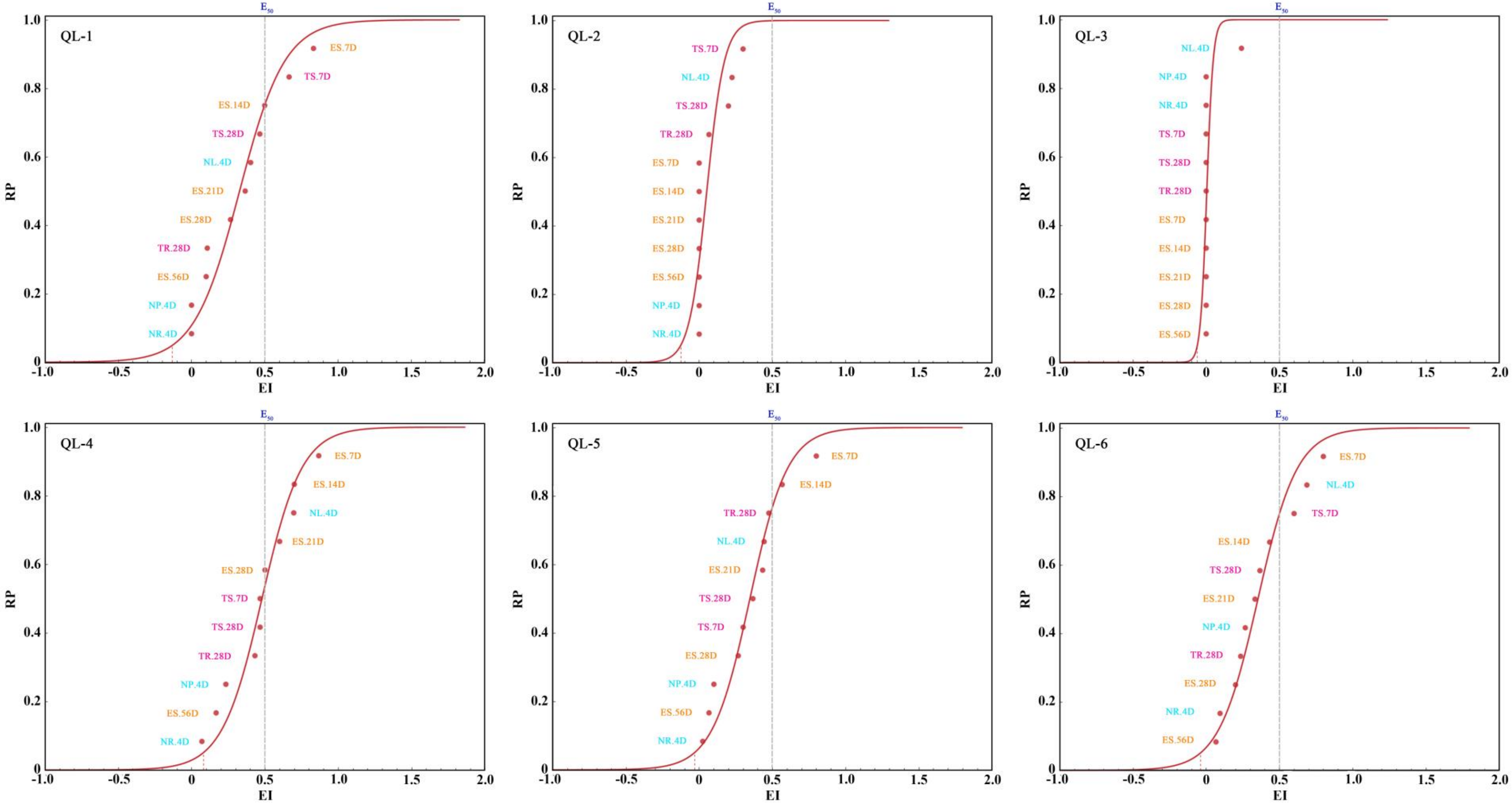

Figure S4. EI cumulative probability distribution of soils collected at QL

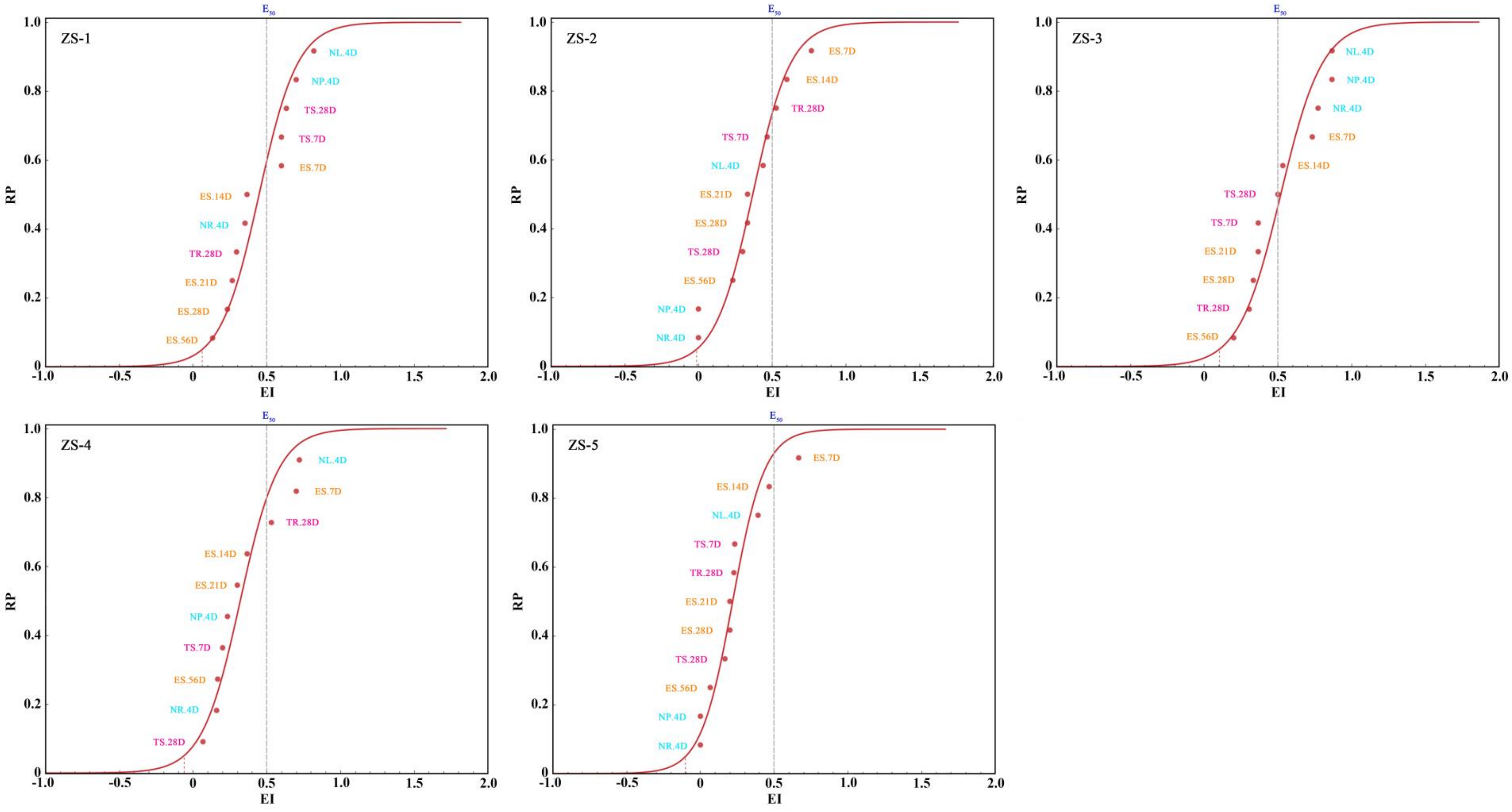

Figure S5. EI cumulative probability distribution of soils collected at ZS

Table S4. Physicochemical properties of soils

Sample point	CEC cmol(+)/kg	pH	OM g/kg	Clay g/kg	Fe mg/kg	Al mg/kg
BY-1	2.8	7.09	33.4	81	29200	4790
BY-2	2.4	8.05	5.8	45	31300	8990
BY-3	9.9	8.07	16.3	224	27200	7570
BY-4	9.1	7.56	115	113	32800	8970
BY-5	4.3	7.50	14.7	67	24400	7880
BY-6	5.4	7.92	12.1	38	26200	7390
DQ-1	9.8	8.32	9	157	18000	8790
DQ-2	9.9	8.53	8.4	215	18200	7740
DQ-3	9.7	8.56	9.6	185	19000	8140
DQ-4	11.9	8.55	6.1	224	16800	9320
DQ-5	9.5	8.33	19.1	164	16900	6430
QL-1	6.1	6.67	22.3	321	62600	15200
QL-2	8.9	4.04	16.7	479	31200	9110
QL-3	4.3	2.62	16.0	113	21800	9940
QL-4	16.9	7.79	45.1	331	24500	10700
QL-5	16.1	7.61	163.0	107	50900	9620
QL-6	4.9	5.97	88.6	350	25500	15800
ZS-1	3.2	6.30	8.0	292	31600	5360
ZS-2	12.1	7.67	23.0	295	42200	9710
ZS-3	6.7	5.59	27.6	418	35200	6310
ZS-4	10.3	7.82	69.5	195	15400	5550
ZS-5	2.7	4.77	6.4	189	24600	6060
